# Experimental jar test dataset for river water clarification using Moringa oleifera

**DOI:** 10.1016/j.dib.2026.113341

**Published:** 2026-10-08

**Authors:** Luis Felipe Nunes Coutinho, Danieli Soares de Oliveira, Clainer Bravin Donadel

**Affiliations:** a Federal Institute of Espírito Santo - campus Vitória, Avenida Vitória, 1.729, 29040-780, Vitória, Espírito Santo, Brazil; b Federal Institute of Espírito Santo - campus Cariacica, Rodovia Governador José Sette, 184, 29150-410, Cariacica, Espírito Santo, Brazil

**Keywords:** Coagulation–flocculation, Natural coagulants, Sustainable treatment technologies, Turbidity removal

## Abstract

This paper presents a comprehensive dataset from laboratory jar test experiments evaluating the application of Moringa oleifera as a natural coagulant for river water clarification, with emphasis on turbidity measurements under different operational conditions. River water samples were subjected to coagulation, flocculation, and sedimentation processes in a controlled laboratory environment using a jar test apparatus. The experiments systematically varied coagulant concentration (40–350 mg/L), mixing speed (82–151 rpm), mixing time (71–450 s), and sedimentation time (0–60 min). Turbidity was measured with a calibrated turbidimeter throughout the clarification process, generating a dataset comprising 3528 experimental records obtained from triplicate tests. The dataset is organized in tabular format and includes operational parameters and turbidity measurements to ensure reproducibility and facilitate data reuse. These data can support comparative studies on natural coagulants, modelling of clarification dynamics, optimization of coagulation–flocculation processes, development of predictive models, and the evaluation of sustainable alternatives to conventional chemical coagulants for water treatment applications.

Specifications Table

**Table:** 

Subject	Earth & Environmental Sciences
Specific subject area	Water treatment using natural coagulants.
Type of data	Table (.xlsx format). Raw data.
Data collection	The dataset was generated under controlled laboratory conditions using a jar test apparatus (Athon, model JTAT6J2LDIG-CS) and a calibrated turbidimeter (AKSO, model TU430). Mature *Moringa oleifera* seeds were peeled, ground into a fine powder, weighed, mixed with tap water under magnetic stirring, and filtered to obtain the liquid natural coagulant. River water samples collected from the study site were subjected to jar test experiments to evaluate turbidity reduction under different operational conditions. Residual turbidity was measured at predefined sedimentation times (0–60 min) for different coagulant concentrations, with all experiments performed in triplicate to ensure reproducibility*.*
Data source location	IFES - Federal Institute of Espírito Santo (Brazil).
Data accessibility	Repository name: Dataset on River Water Clarification Using Moringa oleifera as a Natural Coagulant Data identification number: 10.5281/zenodo.21652410 Direct URL to data: https://doi.org/10.5281/zenodo.21652410
Related research article	None.

## 1 Value of the Data

• This dataset provides a large collection of turbidity measurements obtained from laboratory clarification tests using river water and Moringa oleifera as a natural coagulant, generating reproducible data under controlled operating conditions.
• The experimental records cover a broad range of coagulant concentrations, mixing speeds, mixing times, and sedimentation periods, allowing the assessment of multiple clarification scenarios.
• Because the experiments were conducted with river water rather than synthetic suspensions, the dataset offers information that more closely represents conditions encountered in practical water treatment applications.
• The dataset is accompanied by detailed operational parameters and experimental metadata, enabling transparent reporting, independent verification, and full replication of the tests.
• The recorded measurements can be used for the development of predictive models, sensitivity analyses, process optimization studies, and investigations of coagulation–flocculation dynamics.
• These data constitute a useful resource for researchers, environmental engineers, and students interested in sustainable clarification technologies, natural coagulants, and data-driven approaches for water treatment studies.

## 2 Background

Access to safe drinking water remains a global challenge, especially where surface water is the main source and often exhibits high turbidity due to suspended solids [1,2]. Clarification commonly relies on chemical coagulants such as aluminum and iron salts, which, although highly effective, may generate non-biodegradable sludge and raise environmental concerns associated with residual metals [3]. Although turbidity is a key parameter for evaluating water clarification, datasets combining variations in coagulant concentration, mixing conditions, and sedimentation time with turbidity measurements are needed to support comparisons and analyses.

Natural coagulants have emerged as sustainable alternatives, with *Moringa oleifera* being one of the most extensively investigated. Its coagulation activity has been associated with water-soluble cationic proteins acting through charge neutralization and adsorption; however, other seed constituents may also contribute, and their roles remain under investigation [4,5]. Owing to its biodegradability, low cost, and availability, *Moringa oleifera* has attracted considerable interest as an environmentally friendly coagulant for water treatment [[6], [7], [8], [9]].

The dataset presented in this article comprises turbidity measurements obtained from bench-scale jar test experiments using river water treated with *Moringa oleifera* under different operational conditions. Its public availability supports reproducible research and facilitates comparative, optimization, and data-driven studies of coagulation–flocculation processes [[10], [11], [12]].

## 3 Data Description

The dataset compiles systematic laboratory measurements obtained from jar test experiments designed to evaluate the application of Moringa oleifera as a natural coagulant for river water clarification. The experimental records capture variations in coagulant concentration, mixing speed, mixing time, sedimentation time, and turbidity measurements collected from replicate tests. These operational parameters were intentionally varied to investigate their influence on the clarification process under controlled laboratory conditions, while other factors such as sample volume and experimental procedures were maintained constantly throughout the study.

The dataset is organized in a structured tabular format, where each row corresponds to a single experimental observation. The resulting database comprises 3528 records covering a wide range of operating conditions, including coagulant concentrations from 40 to 350 mg/L, mixing speeds from 82 to 151 rpm, mixing times from 71 to 450 s, and sedimentation periods from 0 to 60 min. Table 1 summarizes the variables included in the dataset, their units, and their descriptions. This organization facilitates data exploration, statistical analysis, computational modeling, and process optimization studies. To illustrate the dataset structure, representative examples are presented in Table 2, while the complete dataset contains all experimental observations and replicates.

**Table 1:** Variables and descriptions of the turbidity dataset.

Variable	Unit	Description
Sampling date	-	Date of river water sample collection (YYYY-MM-DD).
Mixing speed	rpm	Mixing speed used during the coagulation/flocculation stage. Reported values include 82, 121 rpm, and 151 rpm.
Mixing time	s	Mixing time used during the coagulation/flocculation stage. Reported values range from 71 s to 450 s.
Coagulant concentration	mg/L	Concentration of Moringa oleifera applied during the experiment. Reported values range from 40 to 350 mg/L.
Sedimentation time	min	Time elapsed during quiescent settling before turbidity measurement. Values include 0 (initial turbidity), 10, 20, 30, 40, 50, and 60 min.
Trial number	–	Identifier of the experimental replicate. Each condition was tested in triplicate, labelled as 1, 2, or 3.
Turbidity	NTU	Measured residual turbidity of the water sample after sedimentation. Expressed in Nephelometric Turbidity Units (NTU).

**Table 2:** Example of residual turbidity data at different initial turbidity levels and coagulant dosages.

Sampling date [-]	Mixing speed [rpm]	Mixing time [s]	Coagulant concentration [mg/L]	Sedimentation time [min]	Trial number [-]	Turbidity [NTU]
2025–09–18	82	150	50	0	1	98.5
2025–09–18	82	150	50	0	2	94.6
2025–09–18	82	150	50	0	3	91.0
2025–09–18	82	150	50	10	1	64.2
2025–09–18	82	150	50	10	2	60.3
2025–09–18	82	150	50	10	3	52.9
…	…	…	…	…	…	…
2025–09–19	82	200	50	0	1	97.1
2025–09–19	82	200	50	0	2	97.6
2025–09–19	82	200	50	0	3	94.2
2025–09–19	82	200	50	10	1	78.1
2025–09–19	82	200	50	10	2	78.1
2025–09–19	82	200	50	10	3	83.0
…	…	…	…	…	…	…
2025–09–19	82	200	100	0	1	96.6
2025–09–19	82	200	100	0	2	96.4
2025–09–19	82	200	100	0	3	98.8
2025–09–19	82	200	100	10	1	80.7
2025–09–19	82	200	100	10	2	81.4
2025–09–19	82	200	100	10	3	80.4
…	…	…	…	…	…	…
2025–04–07	121	71	100	0	1	153.0
2025–04–07	121	71	100	0	2	156.8
2025–04–07	121	71	100	0	3	157.9
2025–04–07	121	71	100	10	1	137.4
2025–04–07	121	71	100	10	2	138.1
2025–04–07	121	71	100	10	3	135.3
…	…	…	…	…	…	…
2025–04–07	121	71	200	0	1	147.6
2025–04–07	121	71	200	0	2	145.9
2025–04–07	121	71	200	0	3	149.0
2025–04–07	121	71	200	10	1	143.1
2025–04–07	121	71	200	10	2	143.2
2025–04–07	121	71	200	10	3	137.0
…	…	…	…	…	…	…
2025–04–07	121	71	300	0	1	165.8
2025–04–07	121	71	300	0	2	166.9
2025–04–07	121	71	300	0	3	168.7
2025–04–07	121	71	300	10	1	168.6
2025–04–07	121	71	300	10	2	167.9
2025–04–07	121	71	300	10	3	169.2
…	…	…	…	…	…	…

To provide a concise overview of the turbidity data and their variability, Table 3 presents descriptive statistics for three representative experimental conditions, including the mean, standard deviation (SD), range, and coefficient of variation (CV) calculated from triplicate measurements (n = 3) at each sedimentation time. The selected conditions correspond to mixing speeds of 82 rpm, a mixing time of 250 s, and coagulant concentrations of 100, 125, and 130 mg/L. Fig. 1 provides the graphical representation of the same conditions as a function of sedimentation time (0–60 min), with symbols representing mean turbidity values and error bars indicating ± one standard deviation. Together, Table 3 and Fig. 1 provide numerical and graphical illustrations of selected experimental conditions, serving as representative examples of the turbidity measurements available in the dataset.

**Table 3:** Descriptive statistics of turbidity measurements for three representative experimental conditions.

Experimental condition	Sedimentation time (min)	Mean turbidity (NTU)	SD (NTU)	Range (NTU)	CV (%)
Condition 1 82 rpm, 250 s, 125 mg/L	0	40.13	1.08	38.90–40.90	2.69
	10	38.33	0.15	38.20–38.50	0.40
	20	30.87	1.46	29.50–32.40	4.72
	30	24.17	1.96	22.30–26.20	8.09
	40	21.43	1.62	19.70–22.90	7.54
	50	17.14	0.68	16.52–17.87	3.98
	60	14.63	0.75	13.78–15.19	5.13
Condition 2 82 rpm, 250 s, 100 mg/L	0	29.67	1.53	28.00–31.00	5.15
	10	29.77	0.96	28.90–30.80	3.23
	20	24.03	0.59	23.60–24.70	2.44
	30	18.82	0.83	18.04–19.70	4.43
	40	14.66	0.69	13.88–15.20	4.73
	50	11.47	0.27	11.29–11.78	2.35
	60	9.38	0.55	8.89–9.97	5.82
Condition 3 82 rpm, 250 s, 130 mg/L	0	61.80	1.40	60.80–63.40	2.27
	10	54.33	1.45	53.40–56.00	2.66
	20	39.67	1.70	37.90–41.30	4.30
	30	29.11	2.09	26.70–30.50	7.19
	40	24.60	0.69	23.80–25.00	2.82
	50	21.13	1.27	20.00–22.50	5.99
	60	19.33	0.47	19.00–19.87	2.43

**Fig. 1:**
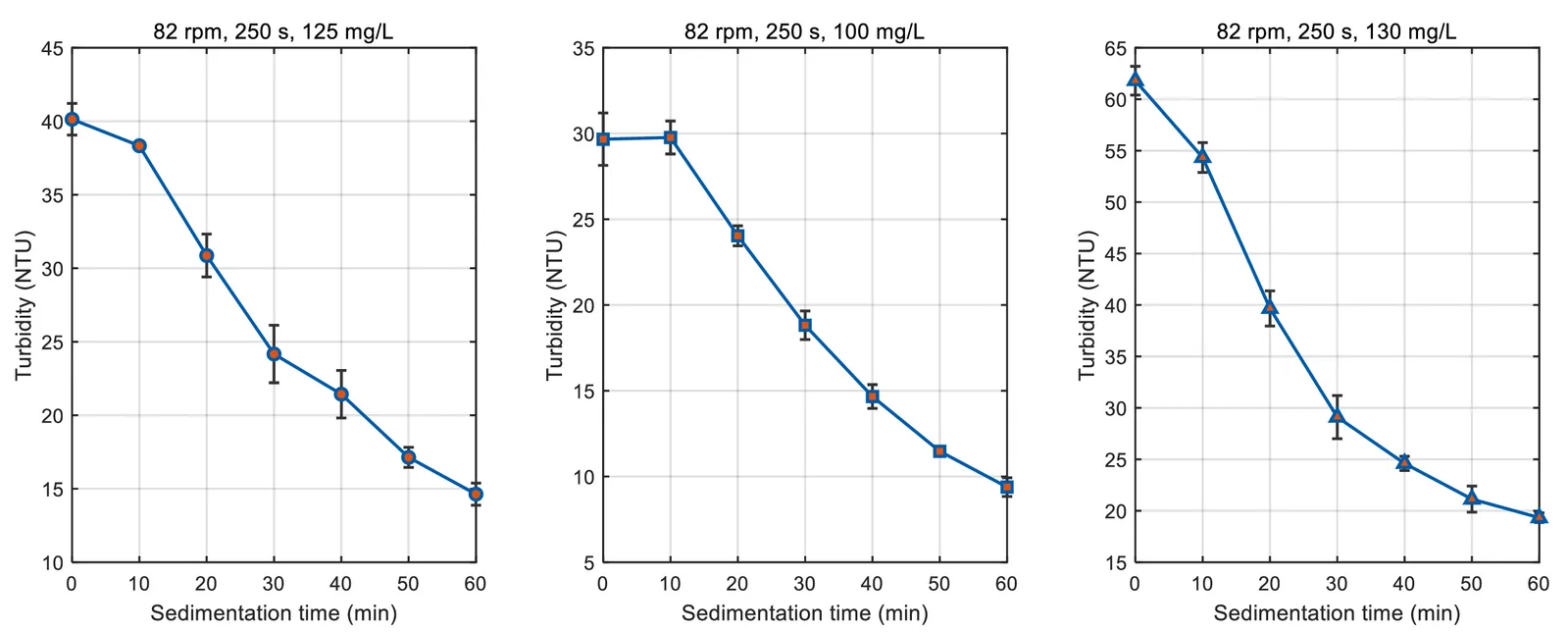
Turbidity as a function of sedimentation time for three representative experimental conditions with coagulant concentrations of 100, 125, and 130 mg/L, at a mixing speed of 82 rpm and a mixing time of 250 s. Symbols represent mean turbidity values, and error bars indicate ± one standard deviation based on triplicate measurements (n = 3).

The complete dataset is available in the Zenodo repository and includes all experimental observations obtained during the study.

## 4 Experimental Design, Materials and Methods

This section describes the procedures adopted to generate the experimental dataset evaluating the application of *Moringa oleifera* as a natural coagulant for river water clarification. The dataset was designed to systematically investigate the influence of operational conditions on turbidity removal using standardized jar test experiments. Turbidity was selected as the response variable because it is one of the principal indicators used to assess the efficiency of coagulation, flocculation, and sedimentation processes in water treatment.

The natural coagulant was prepared from mature *Moringa oleifera* seeds following a standardized protocol [8,9]. The seeds were peeled, ground into a fine powder, and mixed with tap water at a ratio of 1 g of seed powder per 100 mL of water. The mixture was magnetically stirred for 10 min at 500 rpm and then filtered through filter paper with an average pore size of approximately 10 μm to obtain the liquid coagulant used in the experiments (Fig. 2). This standardized procedure ensured the reproducibility and consistency of the generated dataset.

**Fig. 2:**
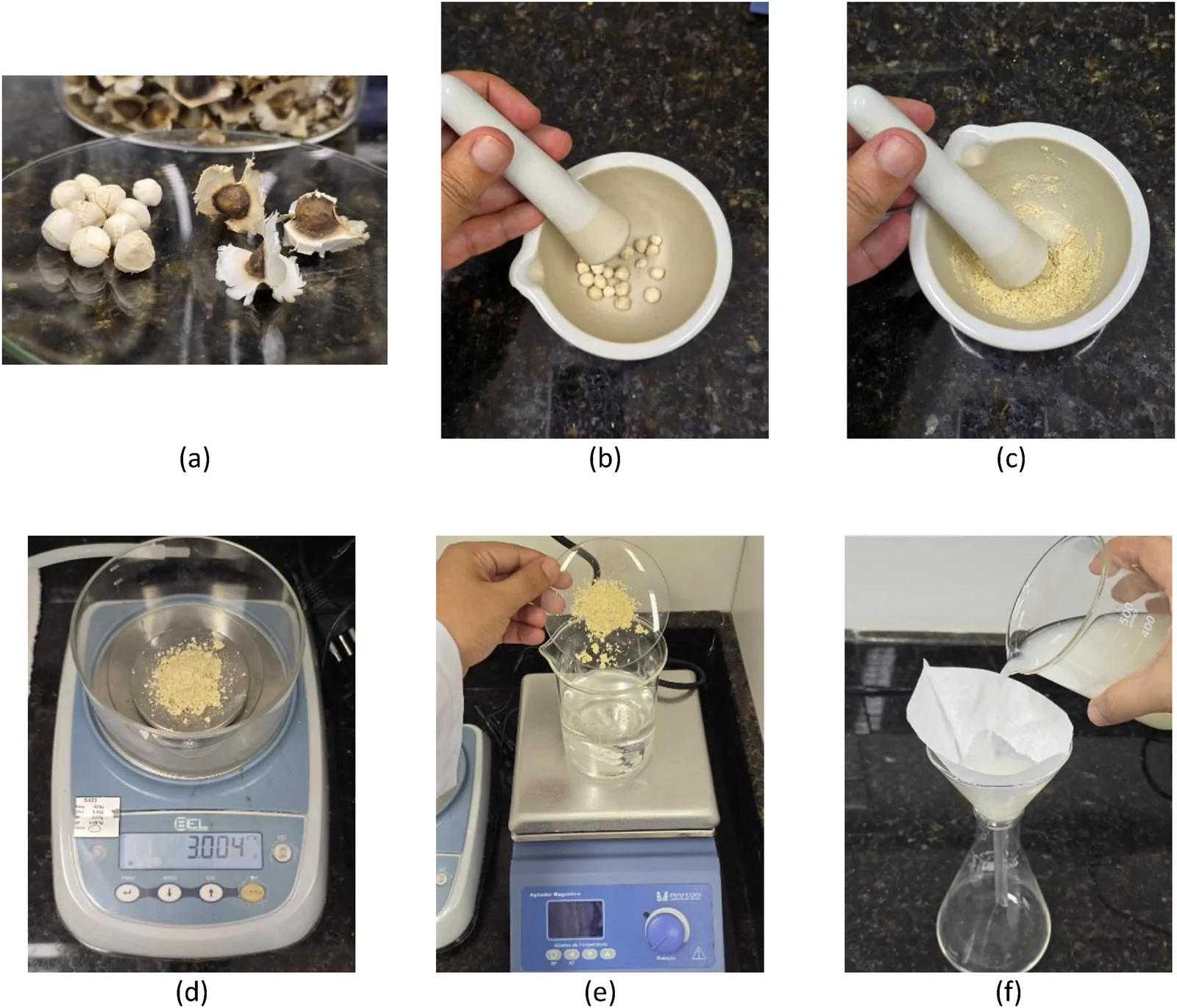
Standardized preparation of the Moringa oleifera-based natural coagulant: (a) peeled seeds; (b) grinding; (c) finely ground seed powder; (d) weighing the powder; (e) mixing with tap water under magnetic stirring; and (f) filtration to obtain the liquid coagulant.

River water samples were collected from the Jucu River (Espírito Santo, Brazil) between March 2025 and March 2026, transported to the laboratory, and stored under refrigerated conditions until testing. Before each experiment, the samples were homogenized and their initial turbidity was measured to characterize the raw water used in the jar test assays. The initial pH of the river water samples was also measured, ranging from 6.6 to 7.9, with a mean value of 7.28 ± 0.35 (n = 168).

Jar test experiments were conducted using a six-place jar test apparatus with 2 L beakers (Fig. 3a), each containing 2 L of river water. The experiments were conducted in a temperature-controlled laboratory maintained at approximately 23 °C. The combinations of coagulant concentration, mixing speed, and mixing time were predefined based on the operational configurations available in the laboratory-scale reactors. These conditions were selected to represent the operating ranges investigated in the experimental assays. The required volume of *Moringa oleifera* coagulant was added to each beaker according to the predefined experimental conditions, followed by coagulation–flocculation under controlled mixing conditions and subsequent sedimentation. Residual turbidity was measured at predetermined time intervals using a calibrated turbidimeter (Fig. 3b). For turbidity measurements, water samples were collected from a fixed sampling point located 7 cm above the bottom of each beaker. All experiments were performed in triplicate, resulting in a dataset containing 3528 experimental records. Each record includes the experimental conditions applied and the corresponding turbidity measurement, providing a structured dataset suitable for comparative studies, process optimization, and predictive modelling.

**Fig. 3:**
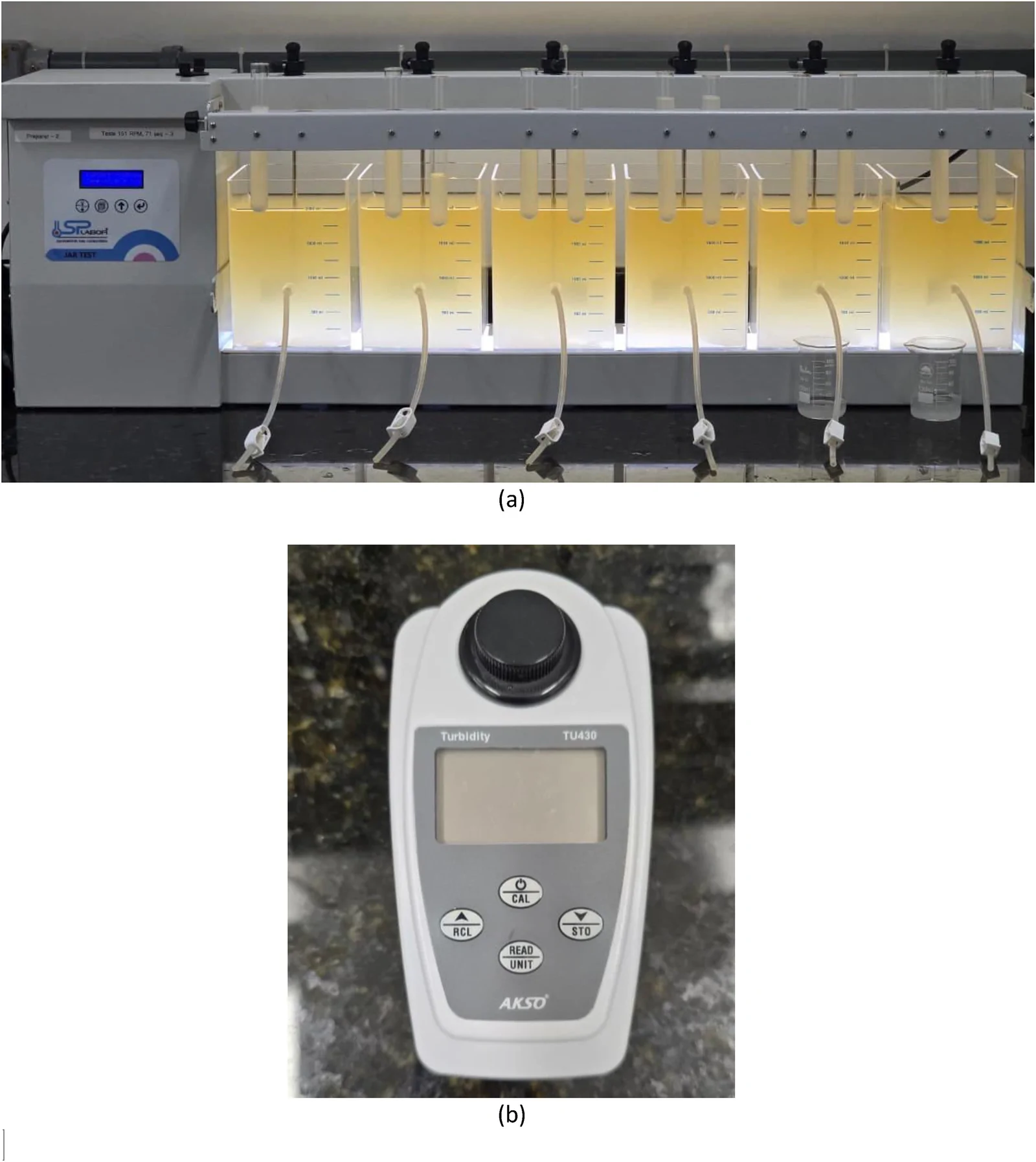
Experimental setup used for the jar test experiments: (a) six-place jar test apparatus and (b) calibrated turbidimeter used for turbidity measurements.

## Limitations

The dataset was generated under controlled laboratory conditions using bench-scale jar test experiments with river water. Therefore, the reported results may not fully represent the hydraulic variability and operational complexity of full-scale drinking water treatment plants.

The dataset includes turbidity measurements collected under predefined experimental conditions and does not encompass additional water quality variables, such as dissolved organic matter, conductivity, alkalinity, or microbiological indicators, which may also influence coagulation performance. Furthermore, the dataset represents short-term clarification experiments and does not address the long-term storage stability of the *Moringa oleifera*-based coagulant or its behavior under continuous-flow operation.

Consequently, the dataset should be interpreted within the experimental conditions investigated and may require additional validation before extrapolation to different water sources or treatment systems*.*

## Ethics Statement

The authors confirm that they have read and comply with the ethical requirements for publication in Data in Brief, and that the current work does not involve human subjects, animal experiments, or any data collected from social media platforms. During the preparation of this manuscript, the authors used artificial intelligence (AI) tools for language editing and improving clarity. The authors reviewed and approved the final content and take full responsibility for this publication.

## CRediT Author Statement

**Luis Felipe Nunes Coutinho**: Data curation, Formal analysis, Investigation, Methodology, Validation, Writing - Original Draft, Writing - Review & Editing **Danieli Soares de Oliveira**: Conceptualization, Formal analysis, Funding acquisition, Investigation, Methodology, Project administration, Supervision, Validation, Writing - Original Draft, Writing - Review & Editing **Clainer Bravin Donadel**: Data curation, Formal analysis, Investigation, Software, Validation, Writing - Original Draft, Writing - Review & Editing.
